# Prediction of Genes That Function in Methanogenesis and CO_2_ Pathways in Extremophiles

**DOI:** 10.3390/microorganisms9112211

**Published:** 2021-10-24

**Authors:** Lulit Tilahun, Asfawossen Asrat, Gary M. Wessel, Addis Simachew

**Affiliations:** 1 Institute of Biotechnology, Addis Ababa University, Addis Ababa P.O. Box 1176, Ethiopia; lulit.tilahun@aau.edu.et (L.T.); addis.simachew@aau.edu.et (A.S.); 2 Department of Mining and Geological Engineering, Botswana International University of Science and Technology, Palapye Private Bag 16, Botswana; kassayea@biust.ac.bw; 3 School of Earth Sciences, Addis Ababa University, Addis Ababa P.O. Box 1176, Ethiopia; 4 Department of Molecular and Cell Biology and Biochemistry, Brown University, Box G, 185 Meeting Street, Providence, RI 02912, USA

**Keywords:** extremophiles, Gaet’ale, Mud’ara, Danakil Depression, Ethiopia syntrophic metabolism, methanogenesis, biotechnology

## Abstract

Gaet’ale (GAL) and Mud’ara (MUP) are two hypersaline ponds located in the Danakil Depression recharged by underground water from the surrounding highlands. These two ponds have different pH, salinity, and show variation in the concentration of many ionic components. Metagenomic analysis concludes that GAL is dominated by bacteria as in the case of the other hypersaline and acidic ponds in the Danakil Depression. However, Archaea dominated the ponds of MUP. In the current study, the application of SEED and KEGG helped to map the ordered steps of specific enzyme catalyzed reaction in converting CO_2_ into cell products. We predict that highly efficient and light-independent carbon fixation involving phosphoenolpyruvate carboxylase takes place in MUP. On the contrary, genes encoding enzymes involved in hydrogenotrophic and acetoclastic methanogenesis appeared solely in ponds of GAL, implying the biological source of the hazardous methane gas in that environment. Based on the investigation of the sources of the genes of interest, it is clear that cooperative interactions between members of the two communities and syntrophic metabolism is the main strategy adapted to utilize inorganic carbon as a carbon source in both MUP and GAL. This insight can be used to design biotechnological applications of microbial communities in production of methane biogas or to minimize CO_2_ emissions.

## 1. Introduction

Microorganisms are fundamental for geochemical cycling and bio-transformation of nutrients in many extreme environments. These extremophilic microorganisms are uniquely adapted to flourish in harsh environments and are particularly important to influence the global bio-geochemical cycling [1,2,3,4,5]. The dynamics between the extreme ecosystems and the extremophiles become more apparent when investigating the responses between biological activities and attributes which characterize an ecosystem [6,7,8,9]. The bio-transformation of minerals and nutrients is highly relevant to understand bio-geochemical cycles in extreme environments. Genomics and physiological adaptations imposed by the extreme conditions on the organisms, are fundamental to understand the interactions between the extreme environments and the organisms [6,7]. Insights into the detailed complexities in the bio-cycle of extreme environments can be revealed using molecular details of microbial pathways coupled with analyses of microbial communities [10,11,12].

The Danakil Depression in northern Ethiopia is a site with numerous polyextreme environments, including the Dallol brines [13], Gaet’ale (GAL) and the Mud’ara ponds (MUP). GAL is an actively bubbling, oily, sulphur-rich cold spring/pool spring, located ~4 km southeast of the Dallol Mountain [14,15]. The GAL Pond, similar to the Black Water Pond, originated from reactivation of a thermal spring during the 2005 major volcano-seismic activity in the Danakil Depression [14,16]. MUP, on the other hand, is an active salt diapir pond south of GAL, with colorful, bubbling cold springs at the northern margin of the hypersaline Lake As’ale, but it has no significant recorded natural history [17]. Excess emission of CO_2_ in GAL has been reported [14] and the consequences of high emanation of CO_2_ might have an impact on the Afar pastoralists and salt miners who frequent the area and, on occasion, tourists. In addition, the pond acts as a water hole for the numerous bird populations in the area.

The importance of CO_2_ is known for plants and microorganisms as the major source of carbon. The accumulation of CO_2_ in the earth’s atmosphere is concerning as it is the major contributor of global warming. In order to solve problems related to CO_2_ emission and industrial application of microorganisms to sequester CO_2_, it is important to investigate the various metabolic potentials of microbes, especially from extreme environments and to engineer them accordingly. Hence, the objective of the study was to investigate the carbon cycle in microorganisms of the GAL acid pond and MUP through in silico metagenome analyses.

## 2. Materials and Methods

### 2.1. Sample Collection and Metagenome Analysis

**Figure 1:**
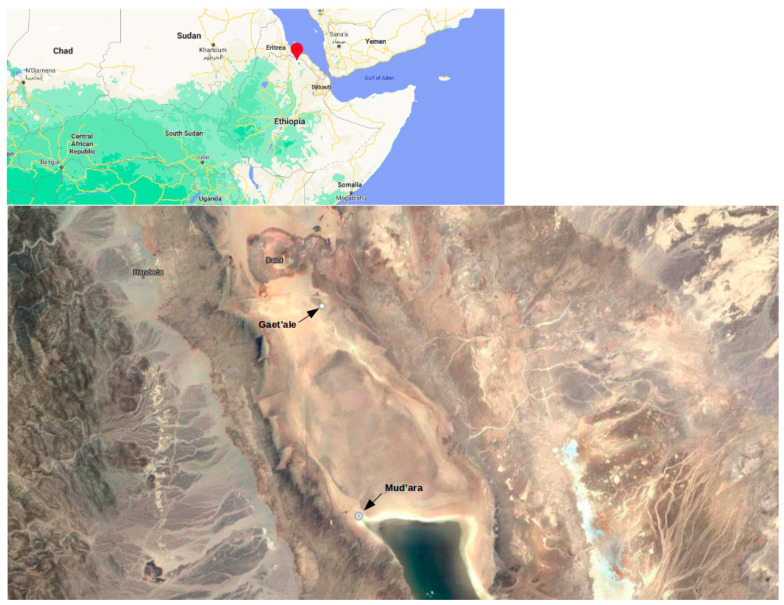
Satellite Image of Gaet’ale and Mud’ara ponds in the northern Ethiopia.

**Figure 2:**
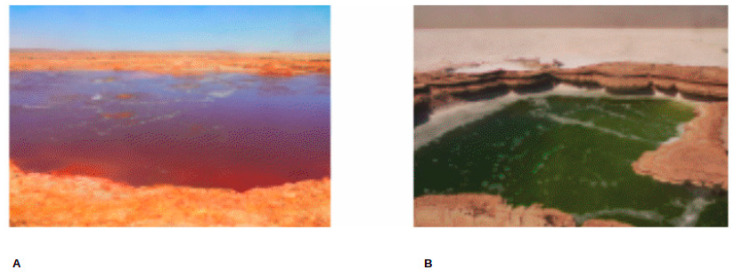
SampSampling sites photos. GAL (**A**) and MUP (**B**) [photo taken by Lulit Tilahun, 2015].

Collection of 2000 mL brine samples each from GAL and MUP ponds was performed on the 4th of February, 2015 at 0642556 E/1571715 N and 0645190E/1558764N, respectively (Figure 1 and Figure 2). The collection and preparation of the Gaet’ale samples for metagenome analysis and physico-chemical analysis and water isotope measurement were carried out as described [13]. Furthermore, for the Mud’ara samples, measurements of pH, conductivity and temperature were performed on-site using portable pH and conductivity meters (430 Enterprise Portable pH and Conductivity Meter 430271, Jenway, Uk). For both sample sites, salinity was measured using a refractometer (DIGIT-0120 ATC, VWR, UK) after diluting the brine samples 1 to 10 times with deionized water. All the brine samples were collected randomly and in triplicates from accessible parts of the ponds considering minimal human and animal contacts to reduce the chances of contamination.

### 2.2. Environmental DNA Extraction and Sequencing

Environmental DNA (eDNA) extraction for Gaet’ale and Mud’ara was accomplished by the DNA extraction method using 1% CTAB-SDS at the Microbial Biotechnology Laboratory (Addis Ababa University) and at the PrIMO Laboratory (Brown University, Providence, RI, USA) as described [18,19]. All eDNA extractions were performed in triplets. To minimize DNA extraction bias, the three replicates of extracted DNA from each sample site were later pooled prior to metagenome sequencing. The quantity and quality of eDNA from all brine samples were checked using the PicoGreen Assay and the Thermo Scientific NanoDrop 3300 Fluorospectrometer. The CovarisTM S220 Ultra-Sonicator was used to fragment the extracted DNA and the TruSeq NANO DNA LT library prep kit (Illumina, San Diego, CA, USA) was used to prepare four dual indexed libraries per the manufacturer’s instructions. Libraries were barcoded and combined into a single group and sequenced on one lane of a flow cell using a 100 bp paired-end run on a HiSeq 2500 instrument (Illumina) at the Genomics Core Facility (Brown University, Providence, RI, USA). Cassava v.2.0, FastQC and Trimmomatic (with Q < 3 and four-base sliding window mean below Q20) were used to demultiplex the sequence run, quality checking sequence composition of the raw data and removing any adapter contamination, respectively.

### 2.3. Assembly and Taxonomic Assignment of Contigs

Quality sequence reads were assembled using metaSPADes with a flag ‘meta’ and kmers 21, 33 and 55 [20]. The resulting metagenome contigs were aligned against the NCBI non-redundant protein database (NCBI-nr) using Double Index Alignment of Next Generation Data (DIAMOND) v0.9.24; Translated Basic Local Alignment Search Tool (BLASTx) with the sensitive mode, frameshift alignment for longer sequences and a default e-value cut-off of 0.001 [21]. The taxonomic assignment of assembled contigs was interactively performed using the MEtaGenome analyzer 6 Community Edition (MEGAN6 CE) [22,23]. Since one contig may contain several Open Reading Frames (ORFs), each ORF was considered separately during the filtration process with alignments that overlapped significantly. Accordingly, MEGAN6 CE assigned the annotated reads onto the NCBI taxonomy tree using settings of the Lowest Common Ancestor (LCA) algorithm for long read adjusted as follows: min score—100.0; max expected—0.01; min percent identity—50; top percent—10 and LCA coverage 80%. The threshold for minimum support that a taxon requires, as a percentage of assigned reads (min support percent), was adjusted to 0.02 so that taxa that obtained at least 0.02% of all aligned bases are reported. As a principle, adjustment of minimum support percent will increase the ‘level of detection” and improve sensitivity for low-abundance species. After the initial automatic binning step, additional manual inspection was performed. Contigs with uncertain taxonomic association, characterized by mixed blastx hits were moved to the ‘Unassigned’ bin. Tree file (“tree.odg”) was exported from MEGAN and uploaded in an online Interactive Tree Of Life (iTOL) version 1.0, for circular phylogenetic tree display [24].

### 2.4. Functional Gene Assignment and Pathway Mapping of Carbohydrate Metabolism

MEGAN6 CE computed SEED RefSeq ids to functional roles using the ‘seed2ncbi.gz’ file from the SEED server for BLAST alignment in NCBI-nr database [22,25,26]. Analysis of functional genes and mapping of carbohydrate metabolism was performed as described [13]. Functional gene evaluation and protein identification for nutrient cycles focusing on carbohydrate metabolism were performed. Contigs with predicted protein-coding genes involved in carbohydrate metabolism were categorized to SEED’s Carbohydrate subsystem. Enzyme Commission (EC) number, or the amino acid sequences of genes (if EC was not available) from SEED categories of key protein families in the Carbohydrate subsystem were retrieved and converted to a KEGG Orthology (KO) identifier. The converted KO identifiers were loaded onto the website “Search & Color Pathway” KEGG Mapping tool on 28/06/2021 (https://www.genome.jp/kegg/tool/map_pathway2.html) for automatic assignment to KEGG’s Metabolism Pathways [27]. Molecular networks and pathway maps of energy metabolism (carbon fixation in photosynthetic organisms and methane metabolism) were in the pathways of carbon fixation and methane metabolism, with well-defined protein families, were further analyzed and compared among GAL and MUP.

## 3. Results

### 3.1. Hydrochemistry

The results of salinity, pH and selected ions measurements are showed in Table 1. GAL is extremely hypersaline and acidic with average percent salinity of 68% and pH ranging between 0 and 1. The measurements of salinity, pH, and temperature indicated that instead, MUP is hypersaline (salinity 36%), slightly acidic (pH: 4.25) and slightly warm (30 °C). The measurement of ion contents showed distinct variation among the two sample sites (Table 1). The Total Phosphorous (TP) recorded in GAL was higher (0.45 g/L) while it was zero in MUP. The quantities of Mg^2+^ and Cl^−^ ions in GAL (19.5 g/L and 432.8 g/L, respectively) were more than the amount in MUP (7.8 g/L and 219.1 g/L, respectively). Chemical oxygen demand (COD) was the highest in GAL and the values of other measured ions (SO_4_^2−^, NO_2_^−^, and NO_3_^−^) are listed in Table 1. The oxygen and hydrogen isotope analysis showed that GAL and MUP are characterized by positive δ^2^H but depleted δ^18^O, attesting to the origin of the brine in these ponds to deeply circulating groundwater generated from meteoric water from cooler, highland sources.

### 3.2. Metagenomics

The total number of reads generated from sequencing is reported in Table 2. The number of sequenced reads generated was 29,741,784 for GAL and 11,016,361 reads for MUP with an average GC content of 52% and 61%, respectively. In general, 100 base pair (bp) long reads were obtained after adapter sequence removal and quality checking. The total contigs generated by MetaSPAdes with a weighted average length for all sample sites are described in Table 2. The length of the shortest contig for which longer and equal length contigs cover at least 50% of the assembly (N50) was 2344 bp for GAL and 383 bp for MUP. Long contiguous bases (more than 429 kilo bases) were obtained from GAL, where the maximum length was a little more than 48 kilo bases. A total of 125.6 Mbp and 22.9 Mbp was assembled for GAL and MUP, respectively, using MetaSPAdes.

**Figure 3:**
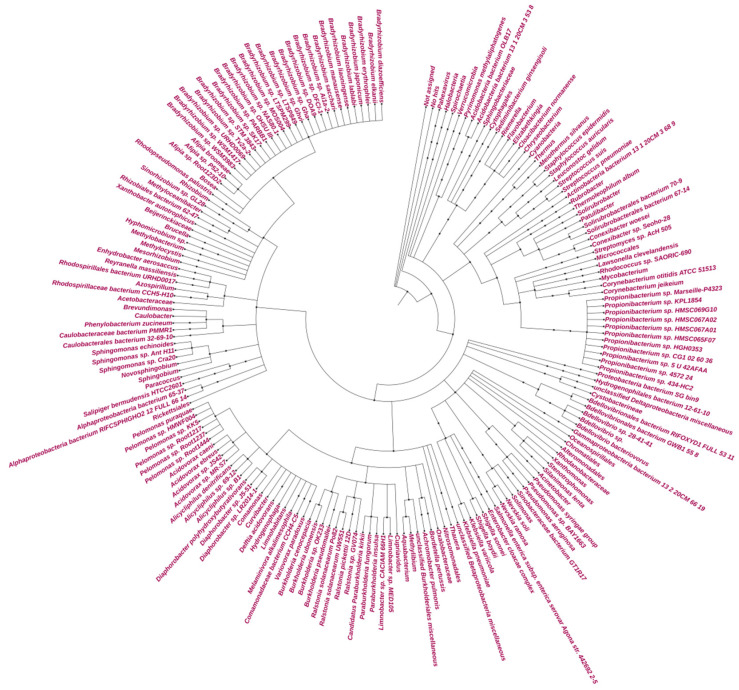
Circular Phylogeny tree for GAL.

**Figure 4:**
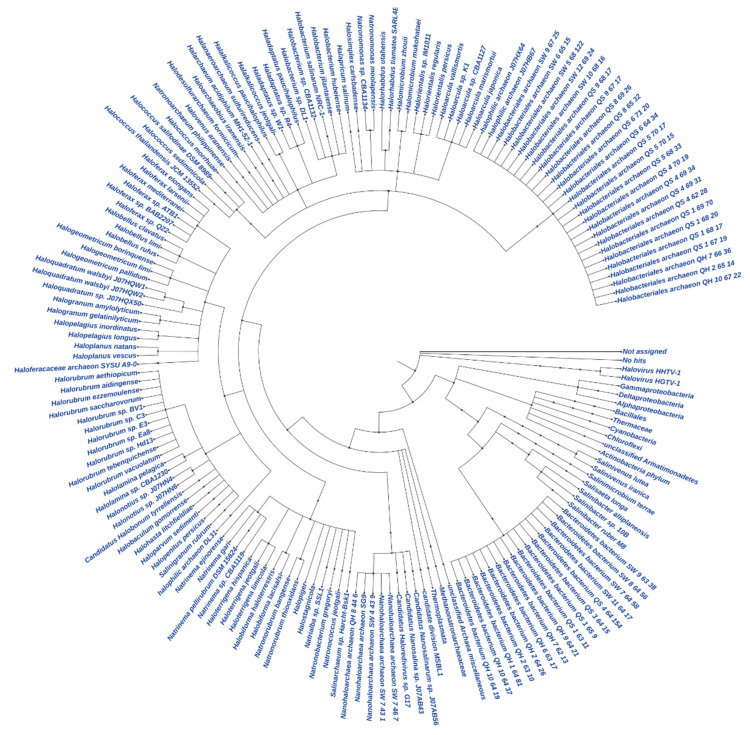
Circular Phylogeny tree for MUP.

The total contigs of more than 76 K and 42 K from GAL and MUP, respectively, were aligned by DIAMOND, which accounted for less than 48% of the contigs from GAL and 71% of the contigs from MUP (Table 3). The result of DIAMOND analysis is considered as an estimation of the taxonomical content (“species profile”) of the sample from which the reads were collected and interactively explored by MEGAN6 CE. Furthermore, the average number of bases per contigs assigned by DIAMOND for GAL was lower than MUP and only 11 million of the bases out of the total 29 million for GAL were assigned to NCBI taxonomy. As a result, the total number of normalized counts of aligned bases assigned to NCBI taxonomy at a species level and the number of OTUs profiled at a species level were less for GAL compared to MUP. In general, no Archaeal OTU was obtained from GAL, while 84% of the total profiled OTUs from MUP were Archaeal at a species level and only 15% were Bacterial. Bradyrhizobium was the most abundant genus in GAL while Halarchaeum was the most abundant genus in MUP (Figure 3 and Figure 4).

### 3.3. Functional Annotation of Reads Based on SEED Database

Important metabolic pathways of the four biogeochemical cycles (C, N, P and S) were identified in GAL and MUP even though the functional annotations of genes were performed for only less than 1/3 of their total metagenomes. The total percentage of functionally annotated reads is less than 18% and 8.5% in GAL and MUP assembled metagenomes, respectively (Table 4). The number of protein coding genes predicted in GAL for Phosphorus metabolism was minimal (0.36% of the total) and only 2.46% and 1.46% of the total predicted genes in GAL were parsed to Nitrogen and Sulfur metabolism. On the other hand, 4.7% of the total predicated genes for MUP were parsed under phosphorous metabolism while only 1.71% and 2% were parsed to Nitrogen and Sulfur metabolisms. In both GAL and MUP, a higher amount of total annotated reads and gene coding ORFs were categorized to subsystems of carbohydrate metabolism. In general, the total amount of predicated protein families was reflected by the percentage of functionally annotated reads.

Rhizobiales, Burholderiales and Propionibacteriales, are among the top five abundant orders in GAL, which are the primary sources of ORFs for predicted protein families in the carbohydrate, nitrogen, phosphorus, and sulfur metabolism and covered approximately 70% of binning to carbohydrate, nitrogen, and sulfur metabolism, while less than 30% was assigned to phosphorous metabolism (Supplementary Table S1). In the case of MUP, more than a quarter of the total assigned bases to carbohydrate, nitrogen, sulfur and phosphorous metabolisms were from the top three abundant orders (Haloferacales, Halobacteriales and Bacteroidales) (Supplementary Table S1).

**Figure 5:**
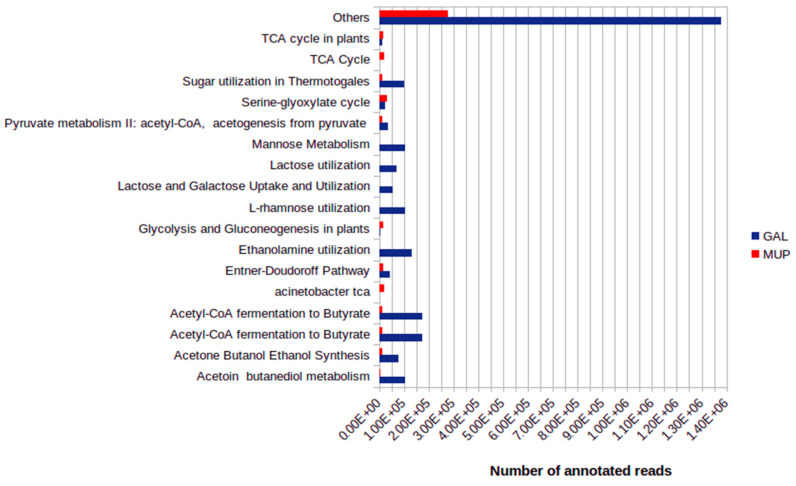
Comparison of major SEED subsystem-connections for carbohydrate metabolism.

Considering only carbohydrate metabolism, approximately 15% and 11% from GAL and MUP metagenomes, respectively, were categorized within the subsystems. Between the two studied sample sites, 109 different subsystems were predicted to be involved in carbohydrate metabolism (Supplementary Table S2). The constructed spreadsheet of subsystems for carbohydrate metabolisms indicated variations between GAL and MUP. For GAL, Acetyl-CoA fermentation to Butyrate, Mannose Metabolism and Ethanolamine utilization were among the top ten major subsystems involved in carbohydrate metabolism while for MUP the major subsystems predicted were Serine-glyoxylate cycle, Acinetobacter TCA and TCA cycle (Figure 5 and Supplementary Table S2).

### 3.4. Mapping of Genes Involved in Carbon Fixation in GAL and MUP

SEED annotated 12 different subsystems involved in carbon fixation in GAL and MUP (Table 5). Only four enzymes were mapped from the GAL samples: (transketolase (EC:2.2.1.1) ribulose-bisphosphate carboxylase large chain (EC:4.1.1.39), ribulose-phosphate 3-epimerase (EC:5.1.3.1), and ribose 5-phosphate isomerase A (EC:5.3.1.6) involved in Reductive pentose phosphate cycle (Calvin cycle). However, more than 25 protein coding genes involved in carbon fixation pathways in prokaryotes (ko00720) and in photosynthetic organisms (ko00710) were mapped for MUP after assignment of KEGG Orthology (KO) numbers (Supplementary Table S3).

Mapping of ‘Reductive pentose phosphate cycle’ for MUP carbon fixation pathways showed that the intermediate Glycerate 3P compound was more likely produced from Glyoxylate and dicarboxylate metabolism than through carbon fixation using the ribulose-bisphosphate carboxylase large chain (EC:4.1.1.39). Instead, enzymes were predicted to be involved in fixation of atmospheric CO_2_ to malate through the dark Crassulacean acid metabolism (CAM) where phosphoenolpyruvate carboxylase (EC:4.1.1.31) fixes CO_2_ to oxaloacetate (Supplementary Figures S1 and S2, Table 6). In addition, all predicated genes involved in the CAM dark reaction were from the taxonomic class of Halobacteriales (Table 6).

### 3.5. Mapping of Methane Metabolism in GAL

Proteins involved in subsystems of “Methanogenesis” and “Methanogenesis from methylated compounds” were mainly predicted in the GAL metagenome (Supplementary Table S4). All of the predicted genes for these subsystems were associated with bacteria, especially with the taxonomic orders Rhizobiales and Burkholderiales (Table 7). Only F420-dependent *N*(5),*N*(10)-methylenetetrahydromethanopterin reductase (EC 1.5.99.11) and *N*(5),*N*(10)-methenyltetrahydromethanopterin cyclohydrolase (EC 3.5.4.27) were absent from GAL’s list of proteins predicated. Hence, complete and/or near complete methane metabolism with four possible methanogenesis pathways were mapped in GAL using KEGG. On the other hand, no protein coding genes for methanogenesis were predicted in the MUP metagenome.

In organisms of the GAL, the first predicted pathway of methanogenesis starts from CO_2_ being reduced to Formyl-MFR by Formylmethanofuran dehydrogenase (EC 1.2.7.12) and subsequently to methane through cascades of catalytic reactions by several enzymes (Supplementary Figure S3). Two enzymes that were not detected in GAL metagenome but vital for this pathway are methenyltetrahydromethanopterin cyclohydrolase (EC 3.5.4.27) and 5,10-methylenetetrahydromethanopterin reductase (EC 1.5.98.2). For the second predicted pathway of methanogenesis, two important enzymes, acetyl-CoA synthetase [EC:6.2.1.1] and acetyl-CoA decarbonylase/synthase, CODH/ACS complex subunit beta [EC:2.3.1.169] were missing (Supplementary Figure S4). The third predicted pathway of methanogenesis is the conversion of methyl-CoM to methane and to methanol. However, the major enzymes involved in conversion of methane to methanol (methane/ammonia monooxygenase [EC:1.14.13.25]) or from methanol to 2-(Methylthio) ethanesulfonate (methyl-Co (III) methanol-specific corrinoid protein], coenzyme M methyltransferase [EC:2.1.1.246]), were not detected (Supplementary Figure S5). For the fourth predicted pathway of methanogenesis, enzymes involved in the dimethylamine and methylamine metabolisms that produces an intermediate methyl CoM were detected (Supplementary Figure S6).

## 4. Discussion

GAL and MUP are two hypersaline ponds among the several found in the Danakil Depression [13,14,15]. The origin of both GAL and MUP is not well investigated but GAL has been in existence at least for the last two decades as it can be recognized in early satellite images [14,15]. What is also clear from the isotopic measurements is the fact that both ponds are fed by meteoric groundwaters which seasonally flow to the basin from the highlands to the west of the Depression. This hydrological link between the highlands and the Danakil Depression has also been established for the adjacent ephemeral Salt Lake As’ale [14]. The difference in the degree of depletion of the δ^18^O (more depleted in GAL than MUP) shows a difference in the input of evaporated water to the ponds. The slightly more enriched δ^18^O in MUP indicates the input of more evaporated water from the adjacent Lake As’ale, which is not the case for GAL.

Previous reports [15,28] gave different results of chemical analysis of the water of GAL. One work [28] reported amounts of Total Organic Carbon (TOC) and SO_4_^2−^ to be 409 ppm (equivalent to COD = 1276.2 ppm [29]) and 117 ppm, respectively, but with no detectable amounts of C, N and S [15]. These results, together with our analysis, indicate the dynamic nature of GAL and the fluctuation of amounts of ions which was possibly influenced by the nearby phreatic eruption on mount Dallol [13,17]. A previous report [14] suggested that high emissions of volcanic derived CO_2_ at GAL could have increased the acidity of the pond. Volcanic derived CO_2_ flow as a supercritical fluid can dissolve in the brine and no longer remain a separate phase [30,31]. Furthermore, hyper-acidic lakes on top of active volcanos are known to trap heat and gas flow (which often includes other gases than CO_2_) originating from deep magmatic intrusions [32]. These two CO_2_ geochemical trapping conditions were observed in GAL hence the reported hazardous gas eruption [14] may not be entirely related to CO_2_. On the other hand, the isotope signatures of the GAL samples are mainly attributed to the water from the highlands through a groundwater connection. In general, our study confirmed that the greasy feeling of the brine from GAL was due to oil as reported [28] as well as supersaturation of dissolved salts [14,15].

Generally, the physico-chemical measurements of the brine samples of GAL and MUP showed variations in the pH, salinity and some ionic contents such as Mg^2+^ and Cl^−^, can influence the diversity of inhabiting microorganisms in the two ponds. MetaSPAdes and MEGAN are among the most reliable and fastest tools for assembling highly uneven metagenomic reads and taxonomic binning, respectively [33]. Assembled contigs from GAL were binned to bacteria, primarily to the phylum Proteobacteria, as in the case of most extreme acidic environments such as Dallol and Black Water [13,34,35,36,37,38]. On the other hand, a large number of assembled contigs from the MUP were binned to Archaea with close to total assignment to the phylum Halobacteria, as in the case of many hypersaline aquatic environments such as Lake Tyrrell in Australia [39].

Many more protein families involved in the nutrient biocycling were identified in GAL compared to MUP. Carbon is the main constituent of living organisms as it is the essential component for all organic polymers [40]. Thus, a large number of contigs with translated ORFs encoding enzymes, transcription factors and different proteins for the carbon metabolism were predicted in this study. According to the KEGG tools, methane metabolism and carbon fixation are grouped under “Energy-Metabolism” as these processes produce the necessary energy. The presence of constraining factors in an ecology such as availability of free oxygen, trace metals and C1 compounds dictate the type of autotrophic pathway in bacteria and archaea [41,42]. Depending on the energy demand of the autotrophic pathways under energy limitation, one or many types of the metabolic pathways (e.g., carbon fixation or methanogenesis) may be adapted by inhabiting organisms [41,42]. The light-independent carbon fixation (dark reaction) was only predicted in MUP while no genes involved in methanogenesis were predicted. This type of carbon fixation especially occurs in lake sediments with low organic matter contents and is mainly facilitated by chemoautotrophic organisms [43]. The prediction of the gene coding for phosphoenolpyruvate carboxylase (EC 4.1.1.31) is the main determinant for mapping the dark carbon fixation pathway in MUP. Hence, the Halobacteria in MUP conceivably assimilates inorganic carbon involving phosphoenolpyruvate carboxylase (EC 4.1.1.31) which participates in capturing inorganic CO_2_ and has a two- to three-fold higher fixation rate than the Calvin cycle [44,45].

For GAL however, several enzymes important for hydrogenotrophic and acetoclastic methanogenesis have been predicted from the metagenome data. Hydrogenotrophic methanogenesis is one of the most primitive of extant metabolisms of respiration where the organisms that use it can grow autotrophically by using H_2_ as electron donor and CO_2_ as sole carbon source and electron acceptor [42,46,47]. The other predicted methanogenesis pathway in GAL is acetoclastic methanogenesis, which is normally activated by the enzyme acetyl-CoA synthetase (EC:6.2.1.1) [48,49]. However, in GAL, instead of acetyl-CoA synthetase, both acetate kinase (EC 2.7.2.1) and phosphate acetyltransferase (EC 2.3.1.8) were mapped in metabolic conversions of acetate to acetyl phosphate and acetyl phosphate to acetyl-CoA, respectively. The actions of both acetate kinase (EC 2.7.2.1) and phosphate acetyltransferase (EC 2.3.1.8) in methanogenesis are usually observed in anaerobic bacteria species and are important for anaerobic decomposition of complex organic matter to methane [50]. In general, as in the case of the acidic West-Siberian peat bog [51], the predicted methane production is likely contributing to the hazardous gas emission in GAL and not to excess CO_2_ as reported [14].

Further investigation on corresponding ORFs for specific enzymes showed different organisms to be the sources of the genes of interest in the carbon fixation and methanogenesis metabolisms. The genes involved in carbon fixation were predicted from Halobacteriaceae and Haloferacaceae while genes involved in methanogenesis were mainly predicted from Bradyrhizobiaceae and Burkholderiacae. A majority of the predicted genes for both types of metabolism have a low percent of identity to their referral genes. The class Halobacteria has been known to have numerous members that are common CO_2_ fixers under light and dark conditions [52]. However, Bradyrhizobiaceae and Burkholderiacaea are the least referred for their methanogenesis capability than other archaeal families [48,50,53,54]. However, while it is well known that most bacteria carry horizontally transferred essential genes that are widespread in Archaea [55], cooperative (syntrophic) interactions among bacteria are also an important survival strategy of microbial consortia in different environments [56,57]. This metabolic cross feeding among microbes enables co-operative growth and metabolic exchange within a shared pool of micronutrients [49,56,57]. In methanogenesis, cooperation of fermentative bacteria to methanogens in basis of the transfer of hydrogen, formate, or acetate to make the degradation of electron-rich substrates thermodynamically favorable was described in [58].

The conversion techniques of carbon dioxide by microbes to its reduced forms such as methane and glucose are important to carbon capture and storage. The application of autotrophic organisms in industries to avoid or minimize CO_2_ emission was not as efficient as needed because of low overall solar-to-product energy conversion efficiencies; hence, mixed-substrate conversions techniques were considered as an option [59,60]. In this study, the predicted genes and metabolic pathways from GAL and MUP indicated that conversion of CO_2_ into biomass or other organic molecules is carried out by groups of different bacterial and archaeal families in their respective communities. The understanding of syntrophic interactions and its contribution in the CO_2_ fixation pathways in the two studied extreme environments can be used as an insight to design industrial as well as environmental biotechnological applications under hypersaline and acidic conditions for either production of methane biogas or to minimize CO_2_ emission from industries. Generally, this result gives insight for future studies that target capturing microbes and genes through synthetic biological approaches.

## 5. Conclusions

Gaet’ale and Mud’ara ponds are two physico-chemically distinct extreme environments found in the Danakil Depression. The CO_2_ geochemical trapping conditions observed in GAL and the predicted methanogenesis pathways in this study, can show that the dangerous condition previously reported might not be entirely caused by a high amount of volcanically generated CO_2_ and is instead caused by a biological origin of methane production. The variation in brine chemistry also influences the diversity of prokaryotes inhabiting these two ponds. The types of autotrophic pathways in MUP and GAL were predicted by the distribution of archaea and bacteria genetic predisposition and possibly influenced by the constraints of their occupied niches. While light-independent carbon fixation by Halobacteria was predicted in MUP, hydrogenotrophic and acetoclastic methanogenesis by Proteobacteria was predicted in GAL. In general, Bradyrhizobiaceae and Burkholderiacae families and Halobacteriaceae and Haloferacaceae families are the main actors of syntrophic interactions in the CO_2_ fixation pathways in GAL and MUP, respectively. This study gives insight for future studies to target microbial communities and/or genes with possible biotechnological potential by designing appropriate mediums and by applications of synthetic biology.

## Figures and Tables

**Table 1:** Hydrogeochemical data of the sample sites.

	GAL	MUP
GPS location (UTM.EW/UTM.NS)	0642556/1571715	0645190/15 58764
Altitude (meter)	−115	−111
Description of sampling sites	Deep orange/oily	Small mixing greenish color pond adjacent to Lake As’ale
Average pH	0 to 1	4.25
Average salinity (%)	68	36
Average temperature	NA	30 °C
Average EC (mS/cm)	NA	235 at 30 °C
Mg^2+^ (g/L)	19.5	7.8
NO_2_^−^ (g/L)	0.12	2.4
NO_3_^−^ (g/L)	0.11	0.13
TP (g/L)	0.45	Nil
COD (g/L)	17.5	1.925
SO_4_^2−^ (g/L)	Nil	0.1
Cl^−^ (g/L)	432.8	219.1
δ^18^O (/mil)	−5.99	−1.38
δ^2^H (/mil)	15.83	3.42

NA: indicates that it was not possible to obtain proper values of temperature, EC and pH. These could not be measured on-site since the instrument was not properly functioning due to the extreme physico-chemical conditions of the pond brine.

**Table 2:** Overview of Metagenomics and assemblies with MetaSPAde.

	**GAL**	**MUP**
Sequence ID	LTW0002	LTW0007
Estimated DNA conc. for sequencing (ng/µL)	2.128	58
Length of single read (bp)	100	100
Total number of reads (Mbp)	29.74	11.01
GC content (%)	52	61
No. of contigs	161,889	59,342
Max. contig length (Kbp)	429.7	48.5
N50 (bp)	2344	383
Total length (Mbp)	125.6	22.9
Metagenome size (bp/ NGS seq, read)	38,505,818/3,480,089	22,881,057/110,16,361

**Table 3:** Taxonomic Binning of assembled reads using DIAMOND and MEGAN6 CE.

	GAL	MUP
No. of bases that must be assigned to taxon/taxa	10,326	2836
Total No. of contigs aligned by DIAMOND	76,755	42,549
Average No. of normalized counts of aligned bases per contig assigned by DIAMOND (bases/contig)	143	236.6
Total No. of normalized counts of aligned bases assigned to NCBI taxonomy (Mb)	11.1	10.1
Total No. of normalized count of aligned bases assigned to NCBI taxonomy at species level (Mb)	3.3	3.6
Total normalized count with no hit	266	715
Total normalized count not assigned (Mb)	3.1	4.1
No. of OTUs profiled at species level	132	154
No. of Archaea	0	128
No. of Bacteria	132	23

**Table 4:** Functional annotation of reads based on SEED database.

	GAL	MUP
Total assigned reads using SEED (absolute)	51.9 Mb	14.2 Mb
Total functionally annotated reads (absolute)	9.2 Mb	1.2 Mb
Total no. of predicted genes	1100	700
Total aligned bases assigned in Carbohydrate metabolism	1.35 Mb	130 Kb
No. of predicted genes in Carbohydrate metabolism	230	150
Total aligned bases assigned in Nitrogen metabolism	165 Kb	10 Kb
No. of predicted genes in Nitrogen metabolism	27	12
Total aligned bases assigned in Phosphorus metabolism	2.86 Kb	29 Kb
No. of predicted genes in Phosphorus metabolism	4	33
Total aligned bases assigned in Sulfur metabolism	138 Kb	15 Kb
No. of predicted genes in Sulfur metabolism	16	14

**Table 5:** SEED functional annotation of aligned bases to subsystems in Carbon fixation.

Subsystems in Carbon Fixation	Total No. of Bases Aligned in Kilo Bases (kb)	
	GAL	MUP
Calvin–Benson cycle	26	4.5
Calvin–Benson–Bassham cycle	25.8	3.1
Carboxysome	34.8	0.4
CO_2_ uptake carboxysome	18.1	1.9
Ethylmalonyl-CoA pathway of C_2_ assimilation	18.5	2.5
Ethylmalonyl-CoA pathway of C_2_ assim., GJO	18.5	2.5
Pentose phosphate pathway	41.3	3.1
Pentose phosphate pathway in plants	40.6	2.7
Photorespiration (oxidative C_2_ cycle)	15.6	10.9
Photorespiration (oxidative C_2_ cycle) plants	32	6.7
TCA Cycle	0.8	19.3
TCA cycle in plants	11.7	16.5
Total No. of protein coding genes	26	39
Total No. of KO	26	38

**Table 6:** ORFs with percentage coverage for protein families in carbon fixation (dark reaction).

EC Number	Enzyme Description	Taxonomic Representation (Family Level)	Coverage (%)	Identity (%)
1.1.1.37	Malate dehydrogenase	Halobacteriaceae	100	91
1.1.1.40	NADP-dependent malic enzyme	Halobacteriaceae	88.4	86
4.1.1.31	Phosphoenolpyruvate carboxylase	Haloferacaceae	8	79

**Table 7:** ORFs with more than 50% coverage for protein families in Methanogenesis and Methanogenesis from methylated compound subsystems.

EC Number	Enzyme Description	Taxonomic Representation (Family Level)	Coverage (%)	Identity (%)
1.8.98.1	CoB-CoM heterodisulfide reductase subunit C	Burkholderiaceae	71	100
	Dimethylamine methyltransferase corrinoid protein	Bradyrhizobiaceae	100	95
	Dimethylamine:corrinoid methyltransferase	Bradyrhizobiaceae	100	91
1.5.99.9	F420-dependent methylenetetrahydromethanopterin dehydrogenase	Burkholderiaceae	18	100
	Formylmethanofuran dehydrogenase (molybdenum) operon gene G	Bradyrhizobiaceae	95	52
1.2.99.5	Formylmethanofuran dehydrogenase (molybdenum) subunit C	Bradyrhizobiaceae	81	86
1.2.99.5	Formylmethanofuran dehydrogenase (tungsten) subunit D	Enterobacteriaceae	50	100
1.2.99.5	Formylmethanofuran dehydrogenase subunit B	Comamonadaceae Bradyrhizobiaceae	59 99.5	99 91
2.3.1.101	Formylmethanofuran–tetrahydromethanopterin N-formyltransferase	Bacteria	100	100
2.8.4.1	Methyl coenzyme M reductase gamma subunit	Comamonadaceae Bradyrhizobiaceae	100 63	100 91
2.8.4.1	Methyl coenzyme M reductase I beta subunit	Burkholderiaceae	90	81
2.8.4.1	Methyl coenzyme M reductase I alpha subunit	Bradyrhizobiaceae	100	83–96
2.8.4.1	Methyl coenzyme M reductase II alpha subunit	Thermaceae	26	97
2.8.4.1	Methyl coenzyme M reductase I gamma subunit	Burkholderiaceae	73	98
2.8.4.1	Methyl coenzyme M reductase II gamma subunit	Burkholderiaceae	41	98
	Monomethylamine methyltransferase corrinoid protein	Bradyrhizobiaceae	97	80
	Monomethylamine permease	Bacilli	100	100
	Monomethylamine:corrinoid methyltransferase	Burkholderiaceae	82	96
	pyrrolysine-containing	Comamonadaceae	98	79.5
2.1.1.86	N5-methyltetrahydromethanopterin:coenzyme M methyltransferase subunit G	Bradyrhizobiaceae	68	79
2.1.1.86	N5-methyltetrahydromethanopterin:coenzyme M methyltransferase subunit H	Rhodothermus	87	76

## Supplementary Materials

The following are available online at https://www.mdpi.com/article/10.3390/microorganisms9112211/s1.
