# Supplementary material for: Prediction of Genes That Function in Methanogenesis and CO2 Pathways in Extremophiles
Source: Microorganisms. 2021 Oct 24;9(11):2211. doi: 10.3390/microorganisms9112211 (PMC8621995; doi:10.3390/microorganisms9112211)
Supplement: Supplementary file 1 [file microorganisms-09-02211-s001.zip › Supplementary Figure S3.pdf]

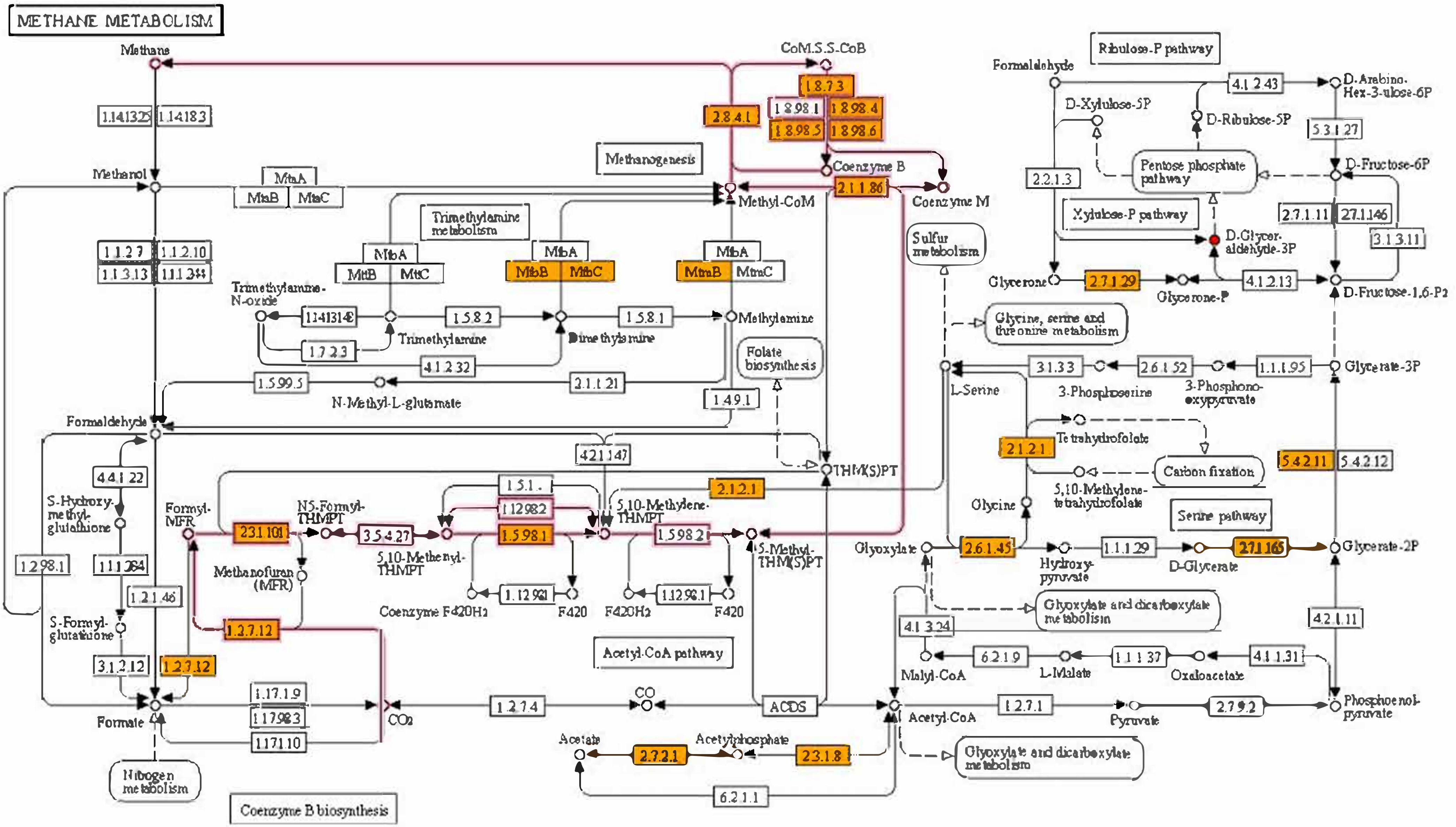

Supplementary Figure S3. 1st predicted pathway of methanogenesis initiated by conversion of CO_2 to Formyl-MFR (pathway mapping produced using SEED-KEGG)
