# Supplementary material for: Prediction of Genes That Function in Methanogenesis and CO2 Pathways in Extremophiles
Source: Microorganisms. 2021 Oct 24;9(11):2211. doi: 10.3390/microorganisms9112211 (PMC8621995; doi:10.3390/microorganisms9112211)
Supplement: Supplementary file 1 [file microorganisms-09-02211-s001.zip › Supplementary table S1.pdf]

Supplementary Table S1. Number of aligned bases assigned in kilo base (kb) to the four nutrient metabolism subsystems of the most abundant taxonomic Order

		Nitrogen	Sulfur	Phosphorous	Carbohydrate
GAL	Burkholderiales	66.8	51.4	0.66	527.3
	Rhizobiales	46.8	51.2	0	293.5
	Propionibacteriales	4.2	6.6	0.2	111.9
	Entrobacteriales	1.9	1.7	0.2	17.7
	Corynebacteriales	0.5	0.25	0	7.9
	others	44.8	26.85	1.8	391.7
MUP	Haloferacales	0.8	0.3	0	9.0
	Halobacteriales	1.9	3.1	6.3	35.6
	Bacteroidetes+	0.3	2.1	1.9	12.7
	Others	7.0	8.5	20.8	72.7
