# Supplementary material for: Prediction of Genes That Function in Methanogenesis and CO2 Pathways in Extremophiles
Source: Microorganisms. 2021 Oct 24;9(11):2211. doi: 10.3390/microorganisms9112211 (PMC8621995; doi:10.3390/microorganisms9112211)
Supplement: Supplementary file 1 [file microorganisms-09-02211-s001.zip › Supplementary Table S2. List of subsystems in Carbohydrate metabolism.pdf]

Supplementary Table 2. List of subsystems in Carbohydrate metabolism		
**Subsystems**	**No. of assigned bases (b)**	
	**GAL**	**MUP**
**2-Ketogluconate Utilization**	4.90E+04	0.00E+00
**Acetoin butanediol metabolism**	1.06E+05	5.16E+03
**Acetone Butanol Ethanol Synthesis**	7.81E+04	1.12E+04
**Acetone carboxylase**	0.00E+00	9.51E+02
**Acetyl-CoA biosynthesis in plants**	1.37E+04	5.23E+03
**Acetyl-CoA fermentation to Butyrate**	1.73E+05	1.31E+04
**acinetobacter tca**	7.98E+02	1.93E+04
**alpha carboxysome**	1.25E+04	4.83E+03
**Alpha-acetolactate operon**	2.37E+03	0.00E+00
**Alpha-Amylase locus in Streptocococcus**	1.51E+04	0.00E+00
**beta carboxysome**	1.79E+04	0.00E+00
**Butanol Biosynthesis**	3.79E+04	8.86E+03
**Calvin-Benson cycle**	2.60E+04	4.61E+03
**Calvin-Benson-Bassham cycle in plants**	2.58E+04	3.07E+03
**Carboxysome**	3.48E+04	4.44E+02
**Chitin and N-acetylglucosamine utilization**	1.42E+04	0.00E+00
**Citrate Metabolism KE**	3.58E+03	4.76E+03
**Citrate Metabolism KE3**	0.00E+00	8.18E+03
**Citrate Metabolism KE4**	9.33E+02	8.39E+03
**CO2 uptake carboxysome**	1.81E+04	1.87E+03
**D-galactarate, D-glucarate and D-glycerate catabolism**	1.93E+03	2.37E+02
**D-galactarate, D-glucarate and D-glycerate catabolism - gjo**	1.93E+03	2.37E+02
**D-galactonate catabolism**	3.77E+03	0.00E+00
**D-Galacturonate and D-Glucuronate Utilization**	3.83E+03	1.19E+03
**D-gluconate and ketogluconates metabolism**	2.48E+03	0.00E+00
**D-ribose utilization**	9.45E+03	2.43E+02
**D-Sorbitol(D-Glucitol) and L-Sorbose Utilization**	3.60E+04	0.00E+00
**D-Tagatose and Galactitol Utilization**	0.00E+00	1.01E+03
**Dehydrogenase complexes**	3.16E+04	5.99E+03
**Deoxyribose and Deoxynucleoside Catabolism**	0.00E+00	1.14E+03
**Dihydroxyacetone kinases**	8.46E+03	0.00E+00
**EC 5.1.3.- Racemases and epimerases acting on carbohydrates and derivatives**	3.89E+03	8.46E+02
**Entner-Doudoroff Pathway**	4.30E+04	1.61E+04
**Ethanolamine utilization**	1.33E+05	0.00E+00
**Ethylmalonyl-CoA pathway of C2 assimilation**	1.85E+04	2.53E+03
**Ethylmalonyl-CoA pathway of C2 assimilation, GJO**	1.85E+04	2.53E+03
**Fermentations in Streptococci**	4.03E+04	3.06E+03
**Fermentations: Lactate**	2.95E+04	1.11E+03
**Fermentations: Mixed acid**	2.81E+04	1.07E+03
**Folate-mediated one-carbon metabolism in plants**	5.61E+02	9.05E+03
**Formaldehyde assimilation: Ribulose monophosphate pathway**	1.24E+04	6.80E+03
**Fructooligosaccharides(FOS) and Raffinose Utilization**	6.69E+03	3.45E+02
**Fructose utilization**	4.06E+03	9.18E+02
**Galactose degradation in plants**	6.52E+03	1.51E+03
**Glycerate metabolism**	2.86E+03	5.10E+02
**Glycerol and Glycerol-3-phosphate Uptake and Utilization, 3-propanediol**	2.19E+03	4.99E+03

Glycerol fermentation to 1	4.66E+04	1.03E+03
Glycogen metabolism	3.09E+03	6.63E+02
Glycolate, glyoxylate interconversions	4.98E+02	0.00E+00
Glycolysis and Gluconeogenesis	1.50E+04	1.19E+04
Glycolysis and Gluconeogenesis in plants	6.52E+03	1.54E+04
Glycolysis and Gluconeogenesis in Streptococci	1.50E+04	7.78E+03
Glycolysis and Gluconeogenesis, including Archaeal enzymes	6.73E+03	1.15E+04
Glycolysis test	7.60E+03	1.16E+04
Glyoxylate bypass	1.65E+02	9.67E+03
Hexose Phosphate Uptake System	4.77E+03	0.00E+00
Inositol catabolism	1.91E+03	0.00E+00
Isobutyryl-CoA to Propionyl-CoA Module	5.52E+04	6.24E+03
L-Arabinose CS	1.86E+04	0.00E+00
L-Arabinose utilization	2.47E+04	0.00E+00
L-ascorbate utilization (and related gene clusters)	2.27E+04	0.00E+00
L-rhamnose utilization	1.07E+05	2.37E+02
Lactate utilization	0.00E+00	7.86E+02
Lactate utilization temp	0.00E+00	1.30E+03
Lacto-N-Biose I and Galacto-N-Biose Metabolic Pathway	0.00E+00	8.46E+02
Lactose and Galactose Uptake and Utilization	5.69E+04	1.11E+03
Lactose utilization	7.20E+04	0.00E+00
Malonate decarboxylase	5.10E+04	0.00E+00
Maltose and Maltodextrin Utilization	2.05E+04	1.68E+03
Mannitol Utilization	1.05E+03	5.34E+02
Mannose Metabolism	1.07E+05	8.76E+02
Methanogenesis	3.57E+04	0.00E+00
Methanogenesis from methylated compounds	1.56E+04	0.00E+00
Methylcitrate cycle	1.95E+04	7.29E+03
Methylglyoxal Metabolism	1.10E+04	5.23E+03
millsd methanogensis	3.57E+04	0.00E+00
N-Acetyl-Galactosamine and Galactosamine Utilization	0.00E+00	5.67E+02
One-carbon metabolism by tetrahydropterines	3.48E+03	1.98E+03
Pentose phosphate pathway	4.13E+04	3.11E+03
Pentose phosphate pathway in plants	4.06E+04	2.68E+03
Peripheral Glucose Catabolism Pathways	1.09E+03	0.00E+00
Photorespiration (oxidative C2 cycle)	1.56E+04	1.09E+04
Photorespiration (oxidative C2 cycle) in plants	3.20E+04	6.68E+03
Propanediol utilization	1.03E+04	2.19E+02
Propionate-CoA to Succinate Module	1.95E+04	7.53E+03
Propionyl-CoA to Succinyl-CoA Module	3.35E+03	1.72E+03
Pyruvate Alanine Serine Interconversions	4.65E+03	2.93E+03
Pyruvate metabolism I: anaplerotic reactions, PEP	1.96E+03	6.06E+03
Pyruvate metabolism I: anaplerotic reactions, PEP in Mycobacteria	2.76E+03	1.06E+04
Pyruvate metabolism II: acetyl-CoA, acetogenesis from pyruvate	3.65E+04	1.33E+04
Pyruvate:ferredoxin oxidoreductase	0.00E+00	7.98E+02
Quinones HGM	0.00E+00	1.11E+04
Rubisco shunt in plants	2.94E+04	4.24E+03
Serine-glyoxylate cycle	2.48E+04	3.10E+04

**Starch biosynthesis in plants**	0.00E+00	6.12E+02
**Starch degradation in plants**	6.52E+03	6.63E+02
**Sucrose metabolism in plants**	8.44E+03	9.58E+03
**Sucrose utilization**	0.00E+00	3.45E+02
**Sugar utilization in Thermotogales**	9.94E+04	1.28E+04
**Tagatose utilization**	0.00E+00	2.64E+02
**TCA Cycle**	7.98E+02	1.93E+04
**TCA cycle in plants**	1.17E+04	1.65E+04
**Trehalose metabolism in plants**	6.52E+03	6.63E+02
**Trehalose Biosynthesis**	6.72E+03	1.28E+03
**Trehalose Uptake and Utilization**	4.66E+04	0.00E+00
**Tricarballylate Utilization**	8.27E+03	0.00E+00
**Unknown carbohydrate utilization (cluster Yeg)**	0.00E+00	5.97E+02
**Xylose utilization**	9.69E+03	4.47E+02
**Xylose utilization in plants**	0.00E+00	2.13E+02
