# Supplementary material for: Prediction of Genes That Function in Methanogenesis and CO2 Pathways in Extremophiles
Source: Microorganisms. 2021 Oct 24;9(11):2211. doi: 10.3390/microorganisms9112211 (PMC8621995; doi:10.3390/microorganisms9112211)
Supplement: Supplementary file 1 [file microorganisms-09-02211-s001.zip › Supplementary Table S3.pdf]

Supplementary Table S3. Predited

ko00710: carbon fixation in photosynthetic organisms

KO	Enzyme description	EC number	GAL	MUP
K00024	Malate dehydrogenase (MDH)	EC:1.1.1.37	√	√
K00134	Glyceraldehyde 3-phosphate dehydrogenase (GAPDH)	EC:1.2.1.12	X	√
K00615	Transketolase (TK)	EC:2.2.1.1	X	√
K00927	Phosphoglycerate kinase (PGK)	EC:2.7.2.3	X	√
K01006	Pyruvate,orthophosphate dikinase (ppdK)	EC:2.7.9.1	X	√
K01624	Fructose-bisphosphate aldolase, class II (FBA)	EC:4.1.2.13	X	√*
K01783	Ribulose-phosphate 3-epimerase (rpe)	EC:5.1.3.1	X	X
K01803	Triosephosphate isomerase (TIM)	EC:5.3.1.1	X	√
K01808	Ribose 5-phosphate isomerase B (rpiB)	EC:5.3.1.6	X	X
K02446	Fructose-1,6-bisphosphatase II (glpX)	EC:3.1.3.11	X	√**
K01601	Ribulose bisphosphate carboxylase large chain	EC 4.1.1.39	√	X
K01602	Ribulose bisphosphate carboxylase small chain	EC 4.1.1.39	√	X
K01672	Carbonic anhydrase	EC 4.2.1.1	√	X

√* Only Fructose-bisphosphate aldolase, class I was detected

√** Only Fructose-1,6-bisphosphatase, type I (EC 3.1.3.11) was detected

ko00720: carbon fixation pathways in prokaryotes

KO	Enzyme description	EC number	GAL	MUP
K00024	Malate dehydrogenase (MDH)	EC:1.1.1.37	√	√
K00031	Isocitrate dehydrogenase (IDH1)	EC:1.1.1.42	X	√
K00031	Isocitrate dehydrogenase (IDH2)	EC:1.1.1.42	X	X
K00174	2-oxoglutarate ferredoxin oxidoreductase subunit alpha (korA)	EC:1.2.7.3	X	X
K00175	2-oxoglutarate ferredoxin oxidoreductase subunit beta (korB)	EC:1.2.7.3	X	X
K00239	Succinate dehydrogenase flavoprotein subunit (sdhA)	EC:1.3.99.1	X	√
K00240	Succinate dehydrogenase iron-sulfur subunit (sdhB)	EC:1.3.99.1	X	√
K00241	Succinate dehydrogenase cytochrome b556 subunit (sdhC)	EC:1.3.99.1	X	X
K00242	Succinate dehydrogenase membrane anchor subunit (sdhD)	EC:1.3.99.1	X	X
K00244	Fumarate reductase flavoprotein subunit (frdA)	EC:1.3.99.1	X	X
K00245	Fumarate reductase flavoprotein subunit (frdB)	EC:1.3.99.1	X	X
K00246	Fumarate reductase subunit C (frdC)	EC:1.3.99.1	X	X
K00247	Fumarate reductase subunit D (frdD)	EC:1.3.99.1	X	X
K00626	Acetyl-CoA C-acetyltransferase (2.AA440)	EC:2.3.1.9	√	√
K00925	Acetate kinase (ackA)	EC:2.7.2.1	√	X
K01006	Pyruvate,orthophosphate dikinase (ppdK)	EC:2.7.9.1	X	√
K01491	Methylenetetrahydrofolate dehydrogenase (NADP+)//Methenyltetrahydrofolate cyclohydrolase (fold)	EC:1.5.1.5 EC:3.5.4.9	X	√
K01679	Fumarate hydratase, class II	EC:4.2.1.2	X	√
K01681	Aaconitate hydratase (ACO)	EC:4.2.1.3	X	√
K01782	3-hydroxyacyl-CoA dehydrogenase//enoyl-CoA hydratase//3-hydroxybutyryl-CoA epimerase (fadJ)	EC:1.1.1.35	√	√
K01847	Methylmalonyl-CoA mutase (MUTB) AA750	EC:5.4.99.2	X	√
K01895	Acetyl-CoA synthetase (ACSS)	EC:6.2.1.1	X	√
K01902	Succinyl-CoA synthetase alpha subunit (sucD)	EC:6.2.1.5	X	√
K01903	Succinyl-CoA synthetase beta subunit (sucC)	EC:6.2.1.5	X	√
K01958	Pyruvate carboxylase (PC)	EC:6.4.1.1	X	√
K01963	Acetyl-CoA carboxylase carboxyl transferase subunit beta (accD)	EC:6.4.1.2	X	X
K01965	Propionyl-CoA carboxylase alpha chain (PCCA)	EC:6.4.1.3	X	X

K01966	Propionyl-CoA carboxylase beta chain (PCCB)	EC:6.4.1.3	X	√
K03518	Carbon-monoxide dehydrogenase small subunit	EC:1.2.99.2	X	X
K03519	Carbon-monoxide dehydrogenase medium subunit	EC:1.2.99.2	X	X
K03520	Carbon-monoxide dehydrogenase large subunit	EC:1.2.99.2	X	X
K05606	Methylmalonyl-CoA/ethylmalonyl-CoA epimerase (MCEE)	EC:5.1.99.1	X	X
K13788	Phosphate acetyltransferase (PTA)	EC:2.3.1.8	√	X
K01644	Citrate lyase beta chain	EC 4.1.3.6	X	√
K00170	Pyruvate:ferredoxin oxidoreductase, beta subunit	EC 1.2.7.1	X	√
K00171	Pyruvate:ferredoxin oxidoreductase, delta subunit	EC 1.2.7.1	X	√
NA	α-ketoglutarate:ferredoxin oxidoreductase	NA	X	X
K00240	Succinate dehydrogenase iron-sulfur protein/ fumarate reductase	EC 1.3.99.1	X	√
NA	2-oxoglutarate ferredoxin:oxidoreductase	EC 1.2.7.3	X	X

Supplementary Table 3. Predited
