# Supplementary material for: Prediction of Genes That Function in Methanogenesis and CO2 Pathways in Extremophiles
Source: Microorganisms. 2021 Oct 24;9(11):2211. doi: 10.3390/microorganisms9112211 (PMC8621995; doi:10.3390/microorganisms9112211)
Supplement: Supplementary file 1 [file microorganisms-09-02211-s001.zip › Supplementary Table S4.pdf]

Supplementary Table S4. Predicte

ko00680 : Methane metabolism

KO	Enzyme description	EC number	GAL	MUP
K03390	CoB--CoM heterodisulfide reductase subunit C	EC 1.8.98.1	√	X
K16179	Dimethylamine methyltransferase corrinoid protein		√	X
K16178	Dimethylamine:corrinoid methyltransferase		√	X
K00319	F420-dependent methylenetetrahydromethanopterin dehydrogenase	EC 1.5.99.9	√	X
K11260	Formylmethanofuran dehydrogenase (molybdenum) operon gene G		√	X
K00202	Formylmethanofuran dehydrogenase (molybdenum) subunit C	EC 1.2.99.5	√	X
K00203	Formylmethanofuran dehydrogenase (tungsten) subunit D	EC 1.2.99.5	√	X
K00201	Formylmethanofuran dehydrogenase subunit B	EC 1.2.99.5	√	X
K00672	Formylmethanofuran--tetrahydromethanopterin N-formyltransferase	EC 2.3.1.101	√	X
K00402	Methyl coenzyme M reductase gamma subunit	EC 2.8.4.1	√	X
K00401	Methyl coenzyme M reductase I beta subunit	EC 2.8.4.1	√	X
K00402	Methyl coenzyme M reductase I gamma subunit	EC 2.8.4.1	√	X
K00399	Methyl coenzyme M reductase II alpha subunit	EC 2.8.4.1	√	X
K00402	Methyl coenzyme M reductase II gamma subunit	EC 2.8.4.1	√	X
K01968	Methylcrotonyl-CoA carboxylase biotin-containing subunit	EC 6.4.1.4	√	X
K20509	Methylmalonyl-CoA decarboxylase, beta chain	EC 4.1.1.41	√	X
K08963	Methylthioribose-1-phosphate isomerase	EC 5.3.1.23	√	X
K16176	Monomethylamine methyltransferase corrinoid protein	NA	√	X
K16238	Monomethylamine permease	NA	√	X
K16176	Monomethylamine:corrinoid methyltransferase	NA	√	X
NA	pyrrolysine-containing	NA	√	X
K00583	N5-methyltetrahydromethanopterin:coenzyme M methyltransferase subunit G	EC 2.1.1.86	√	X
K00584	N5-methyltetrahydromethanopterin:coenzyme M methyltransferase subunit H	EC 2.1.1.86	√	X
K01499	N(5),N(10)-methenyltetrahydromethanopterin cyclohydrolase	EC 3.5.4.27	X	X
